# The interaction between CASK and the tumour suppressor Dlg1 regulates mitotic spindle orientation in mammalian epithelia

**DOI:** 10.1242/jcs.230086

**Published:** 2019-07-15

**Authors:** Andrew P. Porter, Gavin R. M. White, Natalie A. Mack, Angeliki Malliri

**Affiliations:** Cell Signalling Group, Cancer Research UK Manchester Institute, The University of Manchester, Alderley Park, Macclesfield SK10 4TG, UK

**Keywords:** CASK, Dlg1, Mitosis, Mitotic spindle orientation, Oriented cell division, Lumen formation

## Abstract

Oriented cell divisions are important for the formation of normal epithelial structures. Dlg1, a tumour suppressor, is required for mitotic spindle orientation in *Drosophila* epithelia and chick neuroepithelia, but how Dlg1 is localised to the membrane and its importance in mammalian epithelia are unknown. We show that Dlg1 is required in non-transformed mammalian epithelial cells for oriented cell divisions and normal lumen formation. We demonstrate that the MAGUK protein CASK, a membrane-associated scaffold, is the factor responsible for Dlg1 membrane localisation during spindle orientation, thereby identifying a new cellular function for CASK. Depletion of CASK leads to misoriented divisions in 3D, and to the formation of multilumen structures in cultured kidney and breast epithelial cells. Blocking the CASK–Dlg1 interaction with an interfering peptide, or by deletion of the CASK-interaction domain of Dlg1, disrupts spindle orientation and causes multilumen formation. We show that the CASK–Dlg1 interaction is important for localisation of the canonical LGN–NuMA complex known to be required for spindle orientation. These results establish the importance of the CASK–Dlg1 interaction in oriented cell division and epithelial integrity.

This article has an associated First Person interview with the first author of the paper.

## Abstract (precis)

**Highlighted Article:** For mammalian epithelial cells to divide in the correct orientation and form normal luminal structures, Dlg1 must localise to the cell cortex, which is dependent on binding to CASK.

## INTRODUCTION

Control of the orientation of cell division – via correct positioning of the mitotic spindle – plays an important role in developing and maintaining tissue architecture in both embryonic and adult tissues (Bergstralh et al., 2017). Oriented divisions have been observed in a diverse range of organisms and cell types, including yeast, the fruit fly *Drosophila*, chick neuroepithelial and mammalian epithelial systems, and occur in both stem cells and differentiated tissues (di Pietro et al., 2016). Regulated changes in spindle orientation can control the balance between proliferation and differentiation in stratified epithelia (Bergstralh et al., 2013a). The role of spindle orientation in symmetrically dividing epithelial cells is less well understood. Epithelial cells tend to divide in the plane of the epithelium, which is important to integrate daughter cells within the epithelium and thereby maintain barrier function (Bergstralh et al., 2013a). These oriented divisions are hypothesised to have a tumour-suppressive function (Pease and Tirnauer, 2011), although direct evidence of its importance for tumourigenesis is limited at this point.

A conserved set of proteins has been identified that regulate spindle orientation across a range of organisms and tissue types by defining membrane domains for the attachment of astral microtubules, which in turn orient the mitotic spindle (di Pietro et al., 2016). These include Gαi, which is involved in the localisation of leucine-glycine-asparagine (LGN, also known as GPSM2) and subsequently nuclear and mitotic apparatus (NuMA, also known as NUMA1) proteins to the plasma membrane (reviewed in Lu and Johnston, 2013). In turn, NuMA binds to and stabilises astral microtubules, and recruits the microtubule-binding motor protein dynein (Du et al., 2002). In mammalian cells, pulling forces at the cell cortex are transmitted along astral microtubules which then act to correctly position the mitotic spindle (Bergstralh et al., 2017).

In planar divisions of HeLa cells on L-shaped micropatterns, and in chick neuroepithelial cells, LGN localisation is regulated in part by interaction with discs large homolog 1 (Dlg1); LGN membrane localisation is reduced following Dlg1 depletion (Saadaoui et al., 2014), while in *Drosophila* follicular epithelia Dlg1 loss leads to redistribution of Pins (the *Drosophila* orthologue of LGN) (Bergstralh et al., 2013b). However, in other systems, interaction with E-cadherin is required for localisation of LGN (Gloerich et al., 2017). Whether Dlg1 plays a role in orienting the mitotic spindle along the apical–basal axis in non-transformed mammalian epithelial cells has not been determined, and the factor regulating Dlg1 membrane localisation in the context of spindle orientation has yet to be identified (Bergstralh et al., 2017).

In this report we show that Dlg1 is required for spindle orientation in 3D cultures of untransformed mammalian epithelial cells, and identify the membrane-associated guanylate kinase (MAGUK) protein CASK as the protein responsible for Dlg1 membrane localisation in the context of spindle orientation. By blocking CASK–Dlg1 binding we show that this protein–protein interaction is required for Dlg1 localisation, and subsequently the localisation of the LGN–NuMA complex, which binds the astral microtubules that ultimately orient the mitotic spindle. We also show that blocking the CASK–Dlg1 interaction leads to the formation of multilumen structures.

## RESULTS

### Dlg1 regulates spindle orientation and epithelial lumen formation in mammalian cells

**Fig. 1.**
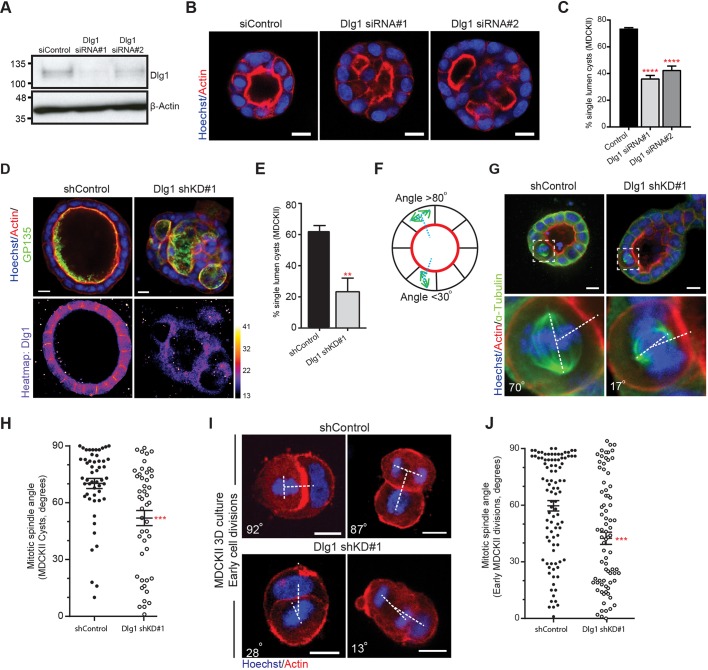
**Dlg1 regulates epithelial lumen formation and mitotic spindle orientation.** (A) Western blot showing depletion of Dlg1 following transfection with two distinct siRNAs. (B) Confocal images of MDCKII cysts transfected with non-targeting siRNA (siControl), or siRNA targeting Dlg1 (siKD#1 and siKD#2), grown in 2% Matrigel, showing multilumen structures, marked with strong actin staining, following Dlg1 depletion. (C) Quantification of single-lumen cysts from three independent Dlg1 knockdown experiments, *n*>40 cysts per experiment; ****P<0.0001 (one-way ANOVA). (D) Confocal images of MDCKII cysts grown in collagen I, constitutively expressing non-targeting shRNA (shControl) or shRNA targeting Dlg1 (Dlg1 shKD#1), stained for the indicated proteins to show lumens (upper panels) or Dlg1 levels (lower panels; heatmap of expression levels, arbitrary units), showing multilumen structures after Dlg1 depletion. (E) Quantification of single-lumen cysts from three independent Dlg1 knockdown experiments as in D, *n*>100 cysts per experiment; **P=0.0083 (unpaired Student's *t*-test, two-tailed). (F) Schematic showing measurement of spindle orientation in MDCKII cysts, showing a normally oriented division (>80°) and a misoriented division (<30°). (G) Representative confocal images of MDCKII cysts grown in collagen I and expressing shControl or Dlg1 shKD#1. Mitotic cells are magnified in inserts (lower panels) and annotated to show mitotic spindle angles. (H) Quantification of spindle angles in constitutive knockdown experiments as in G, *n*=52 and 47 cells pooled from three independent experiments; ***P=0.0004. Data analysed using Mann–Whitney test. (I) Images of early cell divisions (metaphase, left panels; telophase, right panels) in developing MDCKII cysts grown in collagen I. Shown are representative images from shControl and Dlg1 shKD#1 cells, showing misoriented divisions following Dlg1 depletion. (J) Quantification of early cell divisions in MDCKII cysts from experiments as in I, *n*=95 and 78 from three independent experiments; ***P<0.0001 (Mann–Whitney test). Error bars show mean±s.e.m. Scale bars: 10 µm.

MDCKII cells seeded onto Matrigel have the capacity to grow as cysts, reminiscent of those found in the mammalian kidney, with a hollow lumen surrounded by a single layer of epithelial cells. We knocked down Dlg1 using two independent siRNAs (Fig. 1A) and saw that this disrupted normal lumen formation in Matrigel 3D culture, giving rise to cysts with multiple lumens, as marked by strong apical actin staining (Fig. 1B, quantified in C). We next grew cells embedded in a pure collagen I matrix; the cells are entirely surrounded by collagen and so have no external polarity cues, unlike Matrigel culture where they are seeded on a layer of Matrigel under an upper layer of media supplemented with 2% Matrigel. Single MDCKII cells grown for eight to 10 days embedded in this anisotropic collagen I matrix produce cysts with a single lumen, as marked with apical actin and GP135 (podocalyxin) staining (Fig. 1D, top left panel). We grew cells constitutively expressing an shRNA hairpin against Dlg1, which efficiently depleted Dlg1, as shown by the reduction in basolateral staining compared with control cysts (Fig. 1D). Dlg1 depletion led to disrupted lumen development, with many cysts containing multiple lumens (Fig. 1D, quantified in E).

Normal lumen formation in MDCKII cysts has been linked to orientation of the mitotic spindle (Hao et al., 2010; Zheng et al., 2010), so we investigated whether there was a disruption to the angle of the mitotic spindle in MDCKII cells depleted for Dlg1. We measured the angle of divisions in mitotic cells relative to the apical surface of the dividing cell (Fig. 1F). In control cells expressing a non-targeting shRNA the mitotic spindle tended to align orthogonally to the apical surface of the dividing cell, whereas mitotic divisions in Dlg1 knockdown cells were much more widely distributed and essentially randomised (Fig. 1G, quantified in H). This demonstrates a requirement for Dlg1 in spindle orientation in non-transformed mammalian epithelial cells.

Dlg1 is involved in the establishment of polarity in fruit flies (Humbert et al., 2008) and required for adherens junction formation in *C. elegans* (Firestein and Rongo, 2001), and while its role in mammalian epithelial polarity is less clear, if loss of Dlg1 globally affected the polarity of the cyst this might indirectly affect spindle orientation. We therefore investigated spindle orientation in 2D cultures of confluent MDCKII cells, where cells have a strong extrinsic polarity signal from their attachment to the glass coverslip. Control cells aligned their mitotic spindles tightly to the plane of the coverslip, whereas we observed a significant tilting of cell divisions following Dlg1 depletion (Fig. S1A, quantified in B).

Dlg1 localises to lateral cell contacts and therefore loss of Dlg1 may affect spindle orientation through a general defect in cell–cell adhesion. To exclude an indirect effect of Dlg1 via reduced adhesion to adjacent cells we seeded single cells in collagen and measured the orientation of the second division in 3D where cells have only one, apical neighbour (Fig. 1I). In control cells, the mitotic division tended to be orthogonal to the adjacent, apical cell (example image of metaphase and telophase cells in Fig. 1I, quantified in J). Upon knockdown of Dlg1, a randomisation of the angle of cell division was observed (Fig. 1I,J), with many cells dividing directly towards the adjacent cell, indicating that Dlg1 is required for orientation of the mitotic spindle at least in part by cell-autonomous mechanisms independent of lateral cell–cell adhesion.

### CASK is required for Dlg1 membrane localisation, lumen formation and spindle orientation

**Fig. 2.**
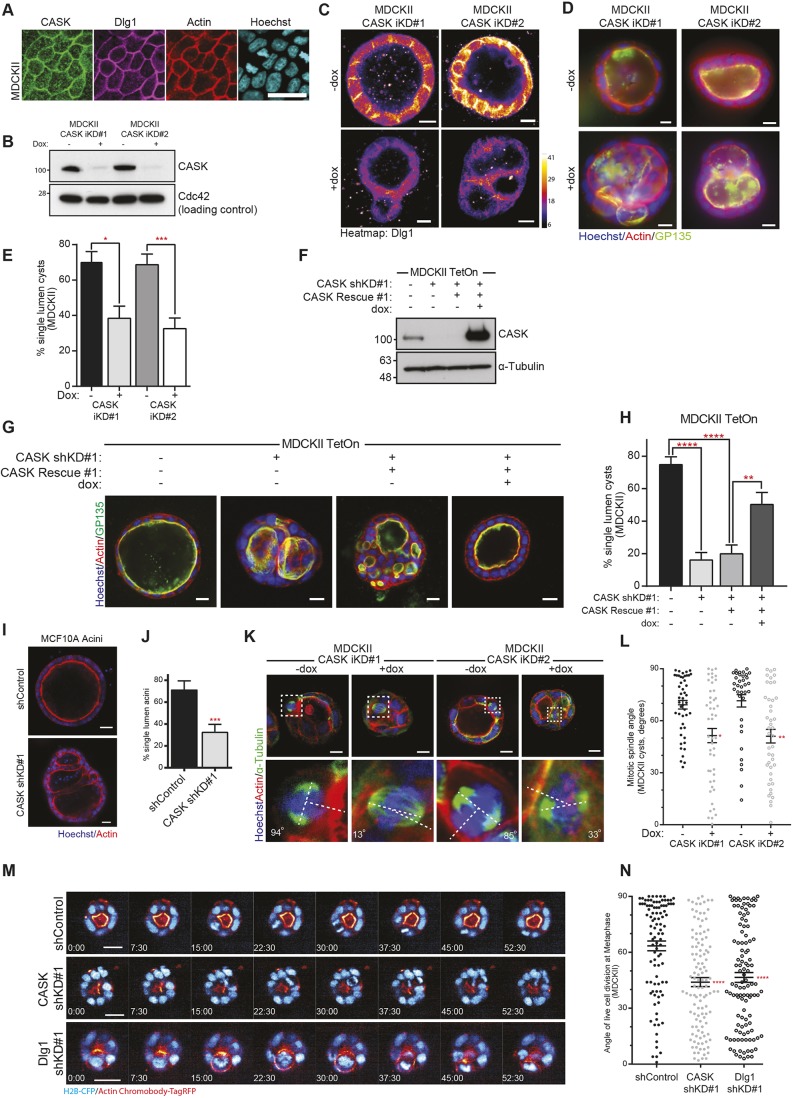
**The Dlg1 interactor CASK is required for normal lumen formation and spindle orientation.** (A) Widefield images showing CASK and Dlg1 colocalisation at the cell cortex in MDCKII cells. Scale bar: 30 µm. (B) Western blot of MDCKII cell lysates from two shRNA doxycycline-inducible knockdown lines (CASK iKD#1 and CASK iKD#2), showing CASK expression in control cells (–dox) and reduction of CASK protein levels following shRNA expression upon treatment with doxycycline (+dox). (C) Confocal images of MDCKII cysts grown in collagen I and stained for Dlg1 protein (heatmap of expression levels, arbitrary units), showing basolateral localisation in control cysts (−dox), and a reduction following CASK knockdown (+dox). (D) Widefield images of MDCKII cysts grown in collagen I show a multilumen phenotype following CASK knockdown upon addition of doxycycline (+dox). Lumen marked with apical actin and GP135 staining. (E) Quantification of single-lumen cysts from experiments as in D. More than 100 cysts examined per experiment. Total number of independent experiments was 6, 6, 8 and 8, respectively; *P=0.0059, ***P=0.0004 (unpaired *t*-test, two-tailed). (F) Western blot of MDCKII cells showing constitutive knockdown of CASK and re-expression of shRNA-resistant wild-type CASK following addition of dox (+dox), restoring CASK protein levels. (G) Confocal images of MDCKII cysts, treated as in F, showing multilumen formation following CASK depletion (−dox), which is rescued by re-expression of CASK (+dox, right panel.) (H) Quantification of single-lumen cysts from experiments as in G with more than 100 cysts examined per experiment. Total number of independent experiments was 6, 3, 5 and 5 respectively; ****P=2.1×10^−5^ for CASK shKD#1 and ****P=7.4×10^−6^ for CASK shKD#1; **P=0.0049 (one-way ANOVA). (I) Confocal images of MCF10A acini grown on 2% Matrigel and stained for actin, showing multi-acini structures following CASK depletion (CASK shKD#1). (J) Quantification of single-lumen MCF10A acini from experiments as in I. *N*=4 independent experiments for both conditions, with more than 100 acini examined per experiment; ***P=0.0003 (unpaired Student's *t*-test, two-tailed). (K) Confocal images of dividing cells in control (−dox) and CASK inducible knockdown cysts (+dox) grown in collagen I; insets show individual divisions annotated to show mitotic spindle angles. (L) Quantification of spindle orientations in control (−dox) and CASK inducible knockdown (+dox) cysts as in K. *n*=47, 48, 35 and 43 cells pooled from three independent experiments; *P=0.0025 for CASK iKD#1 (+dox) and **P=0.0004 CASK iKD#2 (+dox) (Mann–Whitney test). (M) Representative images from movies of dividing cells within MDCKII cysts, grown on Matrigel, showing a normal division in an shControl (non-targeting) cyst (top), and misoriented divisions on CASK and Dlg1 knockdown (middle, CASK shKD#1 and bottom, Dlg1 shKD#1). Time shown in minutes. (N) Quantification of cell division angles from live imaging experiments, only from cells in metaphase. *n*=98, 117 and 112 cells from three independent replicates; ****P=8.2×10^−6^ for CASK shKD#1 and ****P=4.0×10^−5^ for Dlg1 shKD#1 (Kruskal–Wallis test). Error bars show mean±s.e.m. Scale bars: 10 µm.

We next set out to investigate how Dlg1 is localised to the lateral membranes in the context of mitotic spindle orientation. Dlg1 has not been reported to bind directly to cell membranes, and does not contain a membrane localisation domain; instead another factor must be involved in its recruitment to the membrane. It has been reported that the MAGUK protein CASK is involved in Dlg1 localisation to the plasma membrane in some tissues (Lee et al., 2002; Lozovatsky et al., 2009). CASK is a multi-domain scaffolding protein with unusual magnesium-independent kinase activity (Mukherjee et al., 2008) and contains a hook domain, which mediates interaction with the cytoskeleton and allows for membrane binding and basolateral localisation (Cohen et al., 1998; Lee et al., 2002). Like Dlg1, deletion of CASK in mice is lethal (Atasoy et al., 2007), while mutations in CASK are associated with X-linked mental dysfunction and craniofacial abnormalities in humans (Hackett et al., 2010). It is well-characterised in neurons (Hsueh, 2006) where it regulates both trafficking (Lin et al., 2013) and transcriptional pathways (Wang et al., 2004); however, its function in epithelial cells is not understood. We stained 2D cultures of MDCKII cells and saw that CASK colocalised with Dlg1 at cell membranes in interphase and mitosis (Fig. 2A). To test whether CASK was important for Dlg1 localisation we generated MDCKII cells with two different shRNAs targeting CASK mRNA under control of a doxycycline-inducible promoter. Treatment of cells with doxycycline efficiently depleted CASK in cells expressing either shRNA. (Fig. 2B). We next grew these cells as cysts in collagen and stained for Dlg1. In contrast to the clear basolateral staining of Dlg1 seen in the control cells (Fig. 2C, top panels, −dox), basolateral Dlg1 staining is completely lost following CASK knockdown (Fig. 2C, lower panels, +dox). (Of note, we have not been able to successfully image CASK in 3D cultures owing to poor antibody staining in this context.) Additionally, we stained cysts grown in Matrigel, and again observed strong basolateral localisation of Dlg1, which was significantly reduced following CASK depletion (Fig. S2A, quantified in B). Interestingly, other basolateral markers, such as β-catenin, still show a clear basolateral membrane staining in CASK knockdown cysts (Fig. S2C), and individual cells exhibit clear apical–basal polarity with strong apical GP135 staining (Fig. 2D), indicating that loss of CASK does not lead to a general loss of cell polarity that might indirectly account for the loss of Dlg1 localisation.

To test whether CASK and Dlg1 interact in MDCKII cells, we immunoprecipitated endogenous CASK and were able to co-immunoprecipitate endogenous Dlg1 (Fig. S2D). Given that CASK depletion leads to a loss of Dlg1 localisation, we reasoned that CASK depletion alone might be sufficient to disrupt normal lumen formation. We assessed lumen formation in 3D culture using our inducible knockdown cells, and saw that treatment of cells with doxycycline led to a significant decrease in normal cysts, with many more cysts containing multiple lumens compared with untreated cells (Fig. 2D, quantified in E). To demonstrate that CASK depletion itself was responsible for the formation of multiple lumen cysts, we generated a rescue system. First, we produced MDCKII cells with constitutive expression of an shRNA against CASK, which efficiently depleted CASK protein (Fig. 2F). We confirmed that these cells also demonstrated the multiple lumen phenotype (Fig. S2E,F). Next, we used the pRetro-X-Tight dual plasmid system to allow doxycycline-inducible expression of full-length shRNA-resistant CASK. Addition of doxycycline efficiently restored CASK protein expression in these cells (Fig. 2F). In the absence of doxycycline, cells grown in 3D culture produced multilumen structures (Fig. 2G, third panel). Upon addition of doxycycline, CASK expression was restored, and these cells showed a marked decrease in the number of multilumen cysts formed (Fig. 2G, fourth panel, quantified in H). We were able to observe a recovery of Dlg1 membrane localisation in our rescue cells, indicating that CASK itself regulates Dlg1 localisation (Fig. S2G).

CASK is a highly conserved protein (Hsueh, 2006), so we wanted to test whether CASK depletion would have similar effects on lumen formation in other mammalian epithelial cell types. To this end we made use of the normal human breast epithelial cell line MCF10A, which forms acini when grown in Matrigel, with a single hollow lumen surrounded by a layer of epithelial cells. These are similar to the acini found in normal human breast tissue. We infected MCF10A cells with a retroviral plasmid containing a constitutively expressed shRNA against CASK, or a scrambled control (Fig. S2H), and grew the cells in 3D culture. After 10 days of growth, the control cells predominantly formed normal acini with a single lumen (Fig. 2I), whereas CASK knockdown MCF10A frequently formed acini with multiple lumens (Fig. 2I, quantified in J). This demonstrates that CASK is important for lumen formation in non-cancerous human breast epithelial cells.

Next, we tested whether CASK was required for normal spindle orientation. We used our inducible CASK knockdown MDCKII cells and measured mitotic spindle orientation in cysts grown in collagen. The addition of doxycycline, which leads to knockdown of CASK, induced a change in spindle orientation compared with control cysts (-dox), with spindle orientation essentially randomised following CASK depletion (Fig. 2K, quantified in L). We also observed this effect in 2D culture where we saw that the mitotic spindles were tilted away from the plane of the coverslip following constitutive CASK knockdown, similarly to the tilting observed with Dlg1 knockdown (Fig. S2I, quantified in J with the same control as Fig. S1). Next, we determined whether this role for CASK in spindle orientation was conserved in human epithelial cells. We measured the angle of the mitotic spindle in MCF10A cells grown on glass coverslips; in scrambled control cells the mitotic spindle clearly aligned with the plane of the coverslip, whereas in CASK knockdown cells the mitotic spindles became tilted (Fig. S2K,L). These experiments demonstrate a newly discovered role for CASK in epithelial cells, in maintaining epithelial integrity and the orientation of cell division.

Cell division is a dynamic process, the details of which may not be fully captured by snapshots in fixed cells. We therefore developed a method for assaying spindle orientation in live cysts. We transfected MDCKII cells with a nuclear marker (histone-2B CFP) and a marker for actin (actin chromobody tagRFP), allowing us to image the orientation of cell divisions relative to apical surfaces, marked with strong actin staining. We imaged cysts grown on Matrigel using the Opera Phenix High Content imaging microscope, imaging at 7.5 min intervals over periods of 6–12 h. In control cysts (see top panels in Fig. 2M and Movie 1) cells tended to divide in the plane of the epithelium when the angle of cell division was measured at metaphase (Fig. 2N). In cysts with constitutive knockdown of either CASK or Dlg1, we saw a randomisation of the angle of cell division when measured during metaphase (Fig. 2M, quantification in N; see also Movies 2 and 3). As alignment of the mitotic spindle can continue beyond metaphase, there was the possibility that misalignments might be corrected as cell division progressed. We therefore also measured the angle of cell division during anaphase and early cytokinesis. We found that control cells strongly aligned their angle of cell division to the plane of the epithelium during later stages of mitosis (Fig. S2M). In CASK and Dlg1 knockdown cells, while there was a slightly increased population of cells with more aligned spindles than when measured during metaphase, spindle orientation was still significantly misaligned when compared with control cells (Fig. S2M). Together with the results from fixed imaging in both 2D and 3D culture, these findings indicate that both CASK and Dlg1 are required for normal epithelial spindle orientation.

Further, we used our live imaging data to determine the time required for cells to progress from prophase through to early cytokinesis, and found that there was no change in mitotic progression in CASK and Dlg1 knockdown cysts compared to control cysts (Fig. S2N). This indicates that there is no spindle orientation checkpoint in MDCKII cells.

### The direct interaction between CASK and Dlg1 is required for correct spindle orientation

**Fig. 3.**
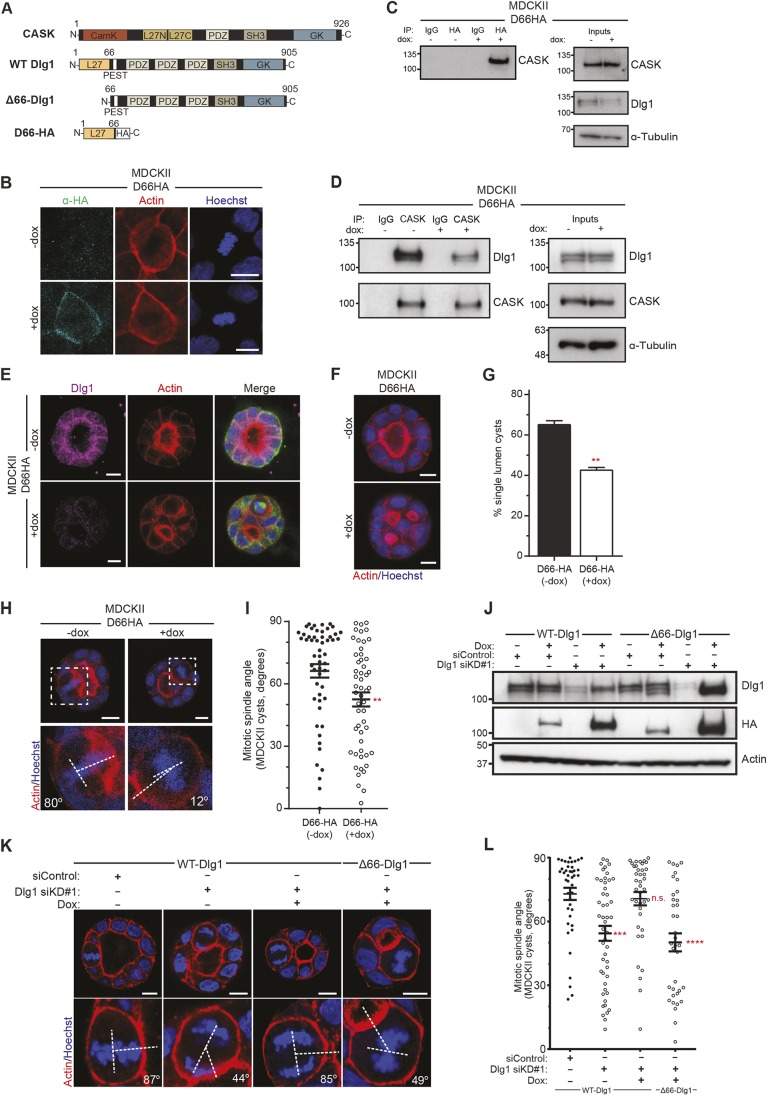
**The interaction between CASK and Dlg1 regulates spindle orientation and lumen formation.** (A) Schematic of the protein structure of Dlg1 and CASK, showing the N-terminal domain of Dlg1 which binds to the L27N domain of CASK. The interfering peptide, D66-HA, is composed of the N-terminal domain of Dlg1 followed by an HA tag; Δ66-Dlg1 lacks the first 66 N-terminal amino acids. (B) Widefield images showing localisation of D66-HA at the cell membrane of metaphase cells when D66-HA is expressed (+dox, bottom panels). (C) Western blot showing an interaction between CASK and D66-HA following HA immunoprecipitation from doxycycline-treated MDCKII lysates expressing the D66-HA interfering peptide. (D) Western blot showing the endogenous interaction between CASK and Dlg1, which is reduced upon expression of D66-HA in MDCKII cells treated with doxycycline (+dox) for 1 day. A representative western blot from three independent experiments is shown. (E) Images of MDCKII cysts grown in Matrigel showing reduction of basolateral Dlg1 staining following expression of D66-HA (+dox). (F) Representative confocal images of MDCKII cysts grown in Matrigel expressing the D66-HA interfering peptide. Doxycycline treatment and subsequent D66-HA expression (+dox) lead to a multilumen phenotype. (G) Quantification of single-lumen cysts following D66-HA expression (+dox). *N*=8 and 9 independent experiments, >100 cysts quantified per experiment; **P=0.002 (Mann–Whitney test). (H) Representative images of metaphase cells in MDCKII cysts with (+dox) or without (−dox) D66-HA expression. Insets annotated to show angle of cell division. (I) Quantification of spindle orientation with and without the expression of D66-HA as in H. *n*=54 and 53 from three independent experiments; **P=0.004 (Mann–Whitney test). (J) Representative western blot showing Dlg1 protein levels in cells treated with control siLuc (siControl) and Dlg1 siKD#1, and re-expression of either siRNA-resistant WT-Dlg1 or Δ66-Dlg1, which restore Dlg1 protein levels. Note that the truncated Δ66-Dlg1 is slightly smaller than WT-Dlg1. (K) Confocal images of mitotic divisions in MDCKII cysts grown in Matrigel, from experiments as in J, showing normal spindle orientation in cells treated with siLuc (siControl), misorientation following Dlg1 depletion (Dlg1 siKD#1), and rescue of spindle orientation with WT-Dlg1 (+dox) but not Δ66-Dlg1 (+dox); insets annotated to show angle of cell divisions. (L) Quantification of spindle orientation from experiments as in K. *n*=42, 49, 39 and 36 mitotic cells across three independent experiments; ***P=0.0003, n.s. *P*=0.94, ****P=0.0001 (one-way ANOVA). Error bars show mean±s.e.m. Scale bars: 10 µm.

Having seen that depletion of either CASK or Dlg1 similarly affected lumen formation and spindle orientation, we set out to investigate further the relationship between these two proteins, and determine whether CASK affects spindle orientation directly through its interaction with Dlg1. The most N-terminal amino acids of Dlg1 comprise an L27 domain [so-called after initial characterisation in the *Caenorhabditis* *elegans* proteins Lin-2 (CASK orthologue) and Lin-7], which mediates intramolecular interactions (Feng et al., 2004). This domain has previously been identified as required for binding to the more N-terminal of the two L27 domains in CASK (Lee et al., 2002) (Fig. 3A).

We reasoned that overexpression of this domain might be sufficient to saturate the Dlg1-binding site on CASK, and thereby block the endogenous CASK–Dlg1 interaction. We generated a doxycycline-inducible plasmid containing the first 66 amino acids of Dlg1 fused to an HA tag (Fig. 3A) and made stable MDCKII cells containing this construct, which we named D66-HA. Similarly to other small interfering constructs we have previously generated (Mack et al., 2012) we were not able to detect the D66-HA peptide directly by western blotting. However, upon treatment with doxycycline, HA expression could be detected at the cell cortex (Fig. 3B), indicating that the construct is expressed and localises similarly to endogenous Dlg1. Next, we tested the capacity of this construct to interact with CASK. We performed an immunoprecipitation in the presence or absence of doxycycline and saw that only in the doxycycline-treated cells was the HA antibody able to co-immunoprecipitate endogenous CASK (Fig. 3C). This indicates that the D66-HA construct is sufficient to interact with CASK in cells. To determine whether expression of the D66-HA construct is able to disrupt normal CASK–Dlg1 interaction, we immunoprecipitated endogenous CASK and probed for Dlg1. In cells treated with doxycycline there was a marked decrease in the amount of Dlg1 immunoprecipitated (Fig. 3D), indicating that expression of the D66-HA peptide is able to block the normal CASK–Dlg1 interaction. We stained MDCKII cysts for Dlg1 and saw that expression of the D66-HA peptide caused a loss of Dlg1 staining at the basolateral membrane in these cysts (Fig. 3E), indicating that reduction in the CASK–Dlg1 interaction interferes with endogenous Dlg1 localisation.

Next, we examined the physiological effects of blocking the CASK–Dlg1 interaction. We grew MDCKII cells containing the D66-HA peptide in 3D culture, and saw that doxycycline treatment increased the number of multilumen cysts compared with untreated cells (Fig. 3F, quantified in G). To test whether the endogenous CASK–Dlg1 interaction is required for normal spindle orientation, we measured the angle of cell divisions in 3D in cells expressing D66-HA, and saw a randomisation of the spindle angle compared with control cysts (Fig. 3H, quantified in I). Expression of this construct was also able to disrupt the orientation of the earliest cell divisions when cells were grown in collagen (Fig. S3A,B), indicating that the CASK–Dlg1 interaction is important even in a context where cells lack lateral neighbours. Taken together, these results indicate that disruption of the direct interaction between CASK and Dlg1 is sufficient to disrupt the basolateral localisation of Dlg1 and interfere with the normal role of these proteins in lumen formation and spindle orientation.

As overexpression of the D66-HA peptide might have off-target effects, we looked to a separate approach to support these findings. We therefore generated MDCKII cells expressing either wild-type Dlg1 (WT-Dlg1) or Dlg1 lacking the first 66 N-terminal amino acids, which contain the CASK-interaction domain (which we named Δ66-Dlg1) (Lee et al., 2002). We confirmed by co-immunoprecipitation that WT-Dlg1 was able to interact with endogenous CASK, whereas we did not detect binding between Δ66-Dlg1 and CASK (Fig. S3C). We depleted endogenous Dlg1 using an siRNA that targets only endogenous Dlg1, and showed by western blotting that we were able to induce near-endogenous levels of the Dlg1 protein constructs on addition of doxycycline to these cells (Fig. 3J). WT-Dlg1 localised primarily to the cell cortex in Dlg1-depleted cells, whereas Δ66-Dlg1 was largely cytoplasmic, supporting a requirement for the interaction with CASK for Dlg1 membrane localisation (Fig. S3D).

To test whether the CASK-interaction domain of Dlg1 is required for normal spindle orientation, we performed a rescue experiment in 3D culture, depleting Dlg1 with siRNA and expressing either WT-Dlg1 or Δ66-Dlg1. Consistent with our results in Fig. 1H, depletion of Dlg1 with siRNA treatment led to a randomisation of the mitotic spindle in 3D culture (Fig. 3K, quantified in L). Re-expression of WT-Dlg1 restored spindle orientation to a similar level to control cysts, whereas re-expression of Δ66-Dlg1 was not able to restore normal spindle orientation (Fig. 3K,L). This demonstrates the specific importance of the N-terminus of Dlg1 in spindle orientation, and supports our findings with D66-HA expression that the specific CASK–Dlg1 interaction is required for normal oriented cell division.

### The CASK–Dlg1 interaction is required for localisation of the canonical LGN–NuMA complex

**Fig. 4.**
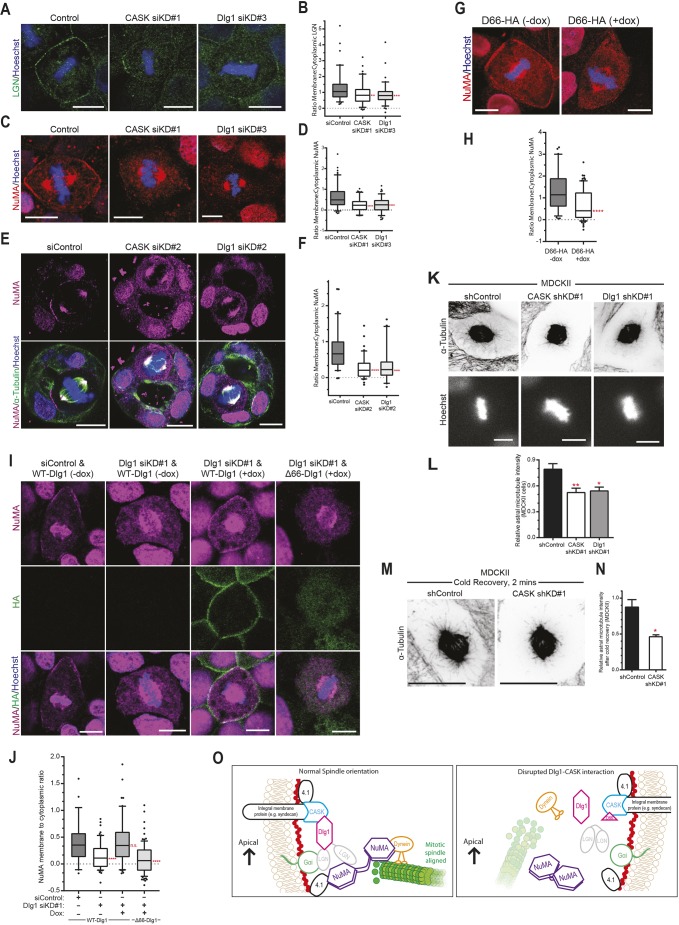
**The CASK–Dlg1 interaction is necessary for the cortical localisation of the spindle orientation complex of LGN and NuMA.** (A) Representative confocal images of LGN staining in control cells (non-targeting siRNA), with reduced membrane-associated LGN in CASK and Dlg1 knockdown cells (CASK siKD#1 and Dlg1 siKD#3). (B) Quantification of membrane:cytoplasmic LGN ratio. *n*=86, 87 and 90 membrane measurements from three independent experiments; **P=0.0013, ***P=0.0005 (one-way ANOVA). Box shows 25–75th percentiles marked with the median, whiskers show 5–95th percentile, dots represent measurements outside this range. (C) Representative confocal images of NuMA staining in control cells (non-targeting siRNA), with reduced membrane-associated NuMA in CASK and Dlg1 knockdown cells (CASK siKD#1 and Dlg1 siKD#3). (D) Quantification of membrane:cytoplasmic NuMA ratio from experiments as in C. *n*=106, 108, and 110 membrane measurements from three independent experiments; ****P=4.9×10^−11^ for CASK siKD#1 and ****P=2.2×10^−10^ for Dlg1 siKD#3 (one-way ANOVA), plotted as in B. (E) Representative AiryScan confocal images of NuMA staining in MDCKII cysts, showing a control siLuc (siControl)-treated metaphase cell with membrane-localised NuMA, and more cytoplasmic NuMA staining following CASK (CASK siKD#2) and Dlg1 (Dlg1 siKD#2) depletion. (F) Quantification of membrane:cytoplasmic NuMA ratio from metaphase cells in 3D culture, as in E. *n*=62, 42 and 40 mitotic cells for control, CASK siKD#2 and Dlg1 siKD#2, respectively, across three independent experiments, plotted as in B; ****P=0.0001, ***P=0.0003 (one-way ANOVA). (G) Confocal images of MDCKII cells in metaphase stained for NuMA, showing a decrease in membrane-localised NuMA upon expression of D66-HA. (H) Quantification of membrane:cytoplasmic NuMA ratio in D66-HA-expressing (+dox) and non-expressing (−dox) cells as in G, *n*=77 and 91 membrane measurements from three independent experiments; ****P=1.4×10^−6^ (unpaired Student's *t*-test, two-tailed), plotted as in B. (I) Confocal images of metaphase MDCKII cells transfected with either siLuc (siControl) or Dlg1 siKD#1, with doxycycline-induced expression of either WT-Dlg1 or Δ66-Dlg1 (+dox), stained for NuMA (magenta) and HA tag (green), showing recovery of cortical NuMA with WT-Dlg1 but not Δ66-Dlg1. (J) Quantification of membrane:cytoplasmic NuMA ratio from metaphase cells in 2D culture from experiments as in I, *n*=57, 60, 100 and 87 mitotic cells across three independent experiments, plotted as in B; ****P=0.0001, n.s. *P*=0.99 (one-way ANOVA). (K) Spinning disc confocal maximum-intensity projections of metaphase cells stained with α-tubulin showing long astral microtubules in control cells (non-targeting shRNA), which are reduced or absent in CASK and Dlg1 knockdown cells (CASK shKD#1 and Dlg1 shKD#1). Images are inverted to more clearly display the astral microtubules. (L) Quantification of astral microtubule intensity, *N*=8, 7 and 4 independent experiments, >20 cells quantified per experiment; **P=0.004, *P=0.0217 (one-way ANOVA). Values are mean±s.e.m. (M) Representative images of MDCKII cells following 2 min recovery from cold treatment. Maximum intensity projections of spinning disc confocal stacks. (N) Quantification of astral microtubule intensities from cold recovery experiments. *N*=3; *P=0.0144 (unpaired Student's *t*-test, two-tailed). Values are mean±s.e.m. (O) Proposed model for the role of the CASK–Dlg1 interaction in localising LGN and NuMA to the lateral membrane and providing a positional cue for the correct orientation of the mitotic spindle. Scale bars: 10 µm.

Spindle orientation requires a conserved set of proteins including LGN, which is normally localised to the basolateral membrane in epithelial cells (Zheng et al., 2010). LGN localisation to the membrane is mediated in part by Gαi binding, but additional factors are also required for correct LGN localisation and function (Seldin and Macara, 2017). We therefore tested whether LGN localisation was affected by depletion of CASK. We stained 2D cultures of MDCKII cells and saw clear membrane localisation of LGN in control metaphase cells, as well as localisation to the spindle poles (Fig. 4A). Upon CASK knockdown, membrane staining of LGN was lost, whereas the spindle pole localisation was unaffected (Fig. 4A). We quantified this effect by measuring the ratio of membrane to cytoplasmic LGN (Fig. S4A), and saw a significant decrease in LGN membrane localisation in CASK-depleted cells (Fig. 4B). Dlg1 has been implicated in LGN localisation in neuroepithelial cells (Saadaoui et al., 2014); we observed a significant decrease in the membrane-to-cytoplasmic ratio of LGN in Dlg1-depleted cells (Fig. 4A,B).

LGN binds to NuMA, and helps localise it to the cell membrane (Du and Macara, 2004; Peyre et al., 2011). We therefore tested whether CASK and Dlg1 are also required for normal NuMA localisation. We stained our 2D MDCKII cultures with a NuMA-specific antibody and in control metaphase cells saw clear staining at the cortex, as well as at the mitotic spindle (Fig. 4C). In both CASK- and Dlg1-depleted cells, we saw a striking reduction in membrane staining of NuMA, while the spindle still stained strongly (Fig. 4C). We quantified the membrane-to-cytoplasmic ratio of NuMA and saw a significant decrease following depletion of either CASK or Dlg1 (Fig. 4D). We were able to restore membrane localisation of NuMA in our CASK rescue cells (Fig. S4B), demonstrating that this effect is specific to loss of CASK. We also observed a decrease in the membrane-to-cytoplasmic ratio of NuMA following siRNA knockdown of CASK and Dlg1 in MCF10A cells in 2D culture (Fig. S4C,D), demonstrating a conserved role for both CASK and Dlg1 in NuMA localisation between mammalian species.

As spindle orientation is of particular importance in a 3D setting for maintenance of lumen formation, we also stained for NuMA in MDCKII cysts grown in Matrigel. We observed strong cortical staining of NuMA in control cells (Fig. 4E), which was significantly reduced following depletion of either CASK or Dlg1 (Fig. 4E, quantified in F), showing that these two proteins are important for NuMA localisation in 3D culture.

To test whether the CASK–Dlg1 interaction itself is required for the correct localisation of NuMA, we first examined cells expressing the interfering D66-HA peptide, and saw a loss of membrane NuMA localisation and a significant reduction in the membrane-to-cytoplasmic ratio of NuMA (Fig. 4G,H). Secondly, we were able to rescue NuMA localisation following Dlg1 depletion in cells expressing WT-Dlg1, but not in cells expressing Δ66-Dlg1, which does not bind CASK or localise to the cortex (Fig. 4I, quantified in J). This indicates that the direct interaction between CASK and Dlg1 is necessary for normal membrane localisation of NuMA, a key component of the spindle orientation machinery.

NuMA is able to interact with microtubules that connect the mitotic spindle to the plasma membrane (Seldin et al., 2016) and provide a pulling force to align the mitotic spindle (di Pietro et al., 2016). We therefore wanted to see whether there was an effect on astral microtubules in cells depleted for CASK or Dlg1. We stained 2D MDCKII cultures for α-tubulin, and imaged metaphase cells to look at astral microtubules. We observed a reduction in astral microtubules in CASK- or Dlg1-depleted cells; we quantified the relative astral microtubule intensity relative to microtubule staining at the mitotic spindle (Fig. S4E) and saw a significant reduction in both CASK- and Dlg1-depleted cells compared with control cells (Fig. 4K, quantified in L). To test whether this was due to a general reduction in astral microtubule numbers, or to an effect on microtubule stability, we subjected cells to cold treatment, which depolymerises microtubules, and then returned them to 37°C to allow microtubule regrowth. A significantly higher regrowth was seen in control cells compared to CASK-depleted cells, indicating a defect in microtubule dynamics following CASK knockdown (Fig. 4M,N), which in turn could explain the inability of the mitotic spindle to correctly orientate.

In summary, our results show that the specific, conserved interaction between CASK and Dlg1 is necessary for cortical recruitment of the spindle orientation machinery in mammalian epithelial cells, and that disruption to this interaction affects the integrity of the epithelial architecture.

## DISCUSSION

Correct spindle positioning is vital for oriented cell division in epithelia (Bergstralh et al., 2017). We have identified a novel role for the MAGUK protein CASK in lumen formation and spindle orientation in epithelial cells. CASK is well-studied in neuronal cells (Hsueh, 2006), and is known to be widely expressed in epithelial cells, although no clear phenotype has previously been associated with epithelial CASK. Our findings here implicate CASK in mitotic spindle orientation through localisation of Dlg1. Downstream of CASK and Dlg1 are the conserved proteins of the spindle orientation complex, LGN and NuMA, whose localisation depends on membrane-bound Dlg1 (see model in Fig. 4O). CASK contains a hook domain that allows for interaction with the membrane via 4.1 proteins as well as by binding to syndecans (Cohen et al., 1998), and therefore provides the link for localisation of Dlg1, which does not itself bind membranes. Loss of CASK phenocopies the effects of directly depleting Dlg1, showing the close relationship between these two proteins.

We used two methods to show that the direct interaction between CASK and Dlg1 is required for normal spindle orientation. Overexpression of the N-terminus of Dlg1 reduces the endogenous CASK–Dlg1 interaction and results in multilumen formation, misoriented cell divisions and mislocalisation of NuMA. Additionally, expressing a truncated mutant of Dlg1, unable to bind to CASK, in cells depleted for endogenous Dlg1 is insufficient to restore normal Dlg1 function. Unlike full-length Dlg1, Δ66-Dlg1 expression is not able to rescue spindle orientation or NuMA localisation. Of note, neither depletion of CASK or Dlg1, nor expression of D66-HA or Δ66-Dlg1 appeared to affect NuMA or LGN localisation to the mitotic spindle.

The D66-HA domain alone localises to the cell cortex, while Dlg1 lacking this domain is largely cytoplasmic, indicating that the N-terminus of Dlg1 is both necessary and sufficient for membrane localisation, and highlighting the importance of the CASK–Dlg1 interaction for the localisation of Dlg1 at the cortex. A recent report demonstrated that the isolated L27 domain of Dlg1 can form homodimers (Ghosh et al., 2018). While our experiments indicate the major role of this domain is the interaction with CASK, the possibility of Dlg1 homodimers could add a further layer of functionality to the multi-protein spindle orientation complex.

Dlg1 has been linked to NuMA localisation at tricellular junctions in fly epithelia (Bosveld et al., 2016), as well as to both LGN and NuMA localisation in HeLa cells (Saadaoui et al., 2014). However, it is apparent that oriented cell divisions are regulated differently in different cell types in different organisms (di Pietro et al., 2016; Saadaoui et al., 2017). Here, we show that this role for Dlg1 is conserved in untransformed mammalian epithelia, both in the well-studied MDCKII dog kidney cell model, and in human breast epithelial cells. Importantly, we show that both CASK and Dlg1 are required for spindle orientation and lumen formation in these two models. This requirement for Dlg1 may explain in part the multilumen phenotype seen in SHIP2-depleted MDCKII cells that also mislocalise Dlg1 (Awad et al., 2013), as well as in prostate epithelial cells depleted of E-cadherin where Dlg1 is lost from the membrane owing to disruption of adherens junctions (Wang et al., 2018). Interestingly, we could see no evidence that Dlg1 itself follows a polarised distribution at the cortex (consistent with previous results in Hart et al., 2017), unlike LGN or NuMA. This indicates that Dlg1 localisation acts as a permissive rather than instructive factor. Therefore, other factors must be required for fine-tuning the localisation or activity of this complex. This may involve other protein–protein interactions mediated by the multi-domain structures of both CASK and Dlg1, which can act as a platform for assembling multi-protein complexes (Wen and Zhang, 2018).

We see a reduction in astral microtubule density following CASK or Dlg1 knockdown, which we hypothesise is due to changes in the rate of capture at the membrane following loss of NuMA localisation. Other studies have reported that changes in astral microtubule number and growth are able to affect spindle orientation; for instance, CYLD stabilises microtubules and promotes assembly of the spindle orientation machinery at the membrane (Yang et al., 2014). Loss of this CASK–Dlg1 complex leading to changes in astral microtubule dynamics suggests the presence of a feedback mechanism that may merit further investigation.

Studies in fruit flies indicate that loss of spindle orientation can be compensated for by reintegration of the misoriented cells back into the epithelium (Bergstralh et al., 2015). Through our live imaging results we saw a range of outcomes for individual misoriented divisions, including reintegration of daughter cells in the epithelium, as well as formation of new luminal structures (Movies 2, 3). However, the resolution of imaging in three dimensions was insufficient to build a quantitative picture of the outcome of these events, and imaging at higher spatial and temporal resolution will be necessary to fully track the outcome of misoriented divisions. Nevertheless, in line with previous reports (Seldin and Macara, 2017), we could detect no spindle orientation checkpoint as oriented and misoriented divisions proceeded at the same rate in our live imaging experiments. This inability of cells to correct misoriented divisions highlights the importance of correctly establishing polarity prior to entry into mitosis.

In humans, mutations in CASK cause mental retardation as a result of microcephaly (Hackett et al., 2010). Microcephaly is a common consequence of misoriented cell divisions in neuronal progenitor cells due to an imbalance in the production of differentiated neurons and self-renewal of progenitors (Lancaster and Knoblich, 2012), and it will be interesting to determine whether the role for CASK in spindle orientation we have uncovered in epithelial cells is conserved in neuronal stem cells. In neurons, CASK binding to Dlg1 alters the conformation of Dlg1, which in turn changes the binding targets of Dlg1 (Lin et al., 2013). While the loss of Dlg1 from the membrane following CASK knockdown may be sufficient to explain the misorientation of the mitotic spindle, it is interesting to speculate that CASK binding may also modify the conformation of Dlg1 to facilitate LGN binding (Saadaoui et al., 2014). Disruption of the CASK–Dlg1 interaction (through loss of the SH3 domain of Dlg1) (Naim et al., 2005) has been reported to impair normal kidney development and lead to the formation of cystic kidneys (Naim et al., 2005); given our results *in vitro* with dog kidney cells in 3D culture, we suspect that this may in part be due to the role of CASK in spindle orientation.

We see that our multilumen structures retain polarity at the cellular level while having lost the overall polarity of the cyst. This is reminiscent of the early stages of many tumours, including ductal carcinoma *in situ* (DCIS) (Debnath and Brugge, 2005), where aberrant structures are contained within a single basement membrane. Dlg1 is one of four mammalian homologues of the *Drosophila* protein Discs large 1 (Dlg1), mutations in which cause tissue overgrowths typical of the loss of a tumour suppressor (Woods and Bryant, 1989). The role of Dlg1 in human disease remains unclear, but immunohistochemistry staining of a variety of tumour types reveals that Dlg1 is often mislocalised to the cytoplasm (reviewed in Roberts et al., 2012). Dlg1 is also the target of viral oncoproteins such the oncogenic human papillomavirus E6, which targets Dlg1 for destruction (Gardiol et al., 1999) and may account for the low levels of Dlg1 observed in cervical cancers. The hepatitis C virus, which is linked to the development of liver cancer, causes a mislocalisation of Dlg1 away from the cell cortex (Awad et al., 2013), which leads to multilumen formation in MDCKII cysts (Awad et al., 2013); our results indicate this is likely to involve misorientation of the mitotic spindle.

Recently, Dlg1 loss has been shown to cooperate with APC mutations to promote intestinal carcinogenesis in a mouse model (Young et al., 2019), an effect thought to be mediated by spindle misorientation due to loss of Dlg1. While a clear causal link between spindle misorientation and tumourigenesis remains elusive (see recent review in Seldin and Macara, 2017), it remains a plausible hypothesis that disruption to the spindle orientation machinery could play a role in tumour initiation or progression. Interestingly, low levels of CASK mRNA are prognostic of poor outcomes in breast cancer (http://kmplot.com/analysis/), indicating a potential role for the CASK–Dlg1 interaction in human disease that merits further investigation.

## MATERIALS AND METHODS

### Antibodies

Antibodies against the following were used for western blotting (WB), immunofluorescence (IF) and immunoprecipitation (IP): α-tubulin (DM1A; Sigma-Aldrich, T6199 mouse; 1:2500 IF, methanol; 1:5000 WB), β-actin (Sigma-Aldrich, clone AC-15 mouse; 1:10,000 WB), Cdc42 (BD Transduction Laboratories, 610928 mouse; 1:1000 WB), GP135/Podocalyxin (gift from Dave Bryant, Beatson Institute for Cancer Research, UK; 1:2000 IF, formaldehyde), CASK (C-6; Santa Cruz Biotechnology, sc-13158 mouse; 1:100 IF; 1:1000 WB; 3 µg IP), Dlg1 (SAP97 H-60; Santa Cruz Biotechnology, sc-25661 rabbit; 1:100 IF, methanol; 1:1000 WB); β-catenin (Upstate, 06-734; 1:200 IF), HA tag (Sigma-Aldrich, 12CA5 mouse; 1:1000 WB), HA tag (Abcam, ab9110 rabbit; 1:2000 WB), HA tag (Roche Diagnostics, 3F10 rat; 1:200 IF, formaldehyde; 1:1000 WB), NuMA [Abcam, ab36999 (now unavailable, replaced with ab109262 in Fig. 4I); 1:150 IF, methanol], LGN (Millipore, ABT-174; 1:250 IF, methanol), pericentrin (Covance, PRB-432C; 1:2000 IF, methanol).

Secondary antibodies and stains used were: IgG peroxidase-conjugated anti-mouse IgG from sheep (GE Healthcare, NA931), anti-rabbit IgG from donkey (GE Healthcare, NA934) and anti-rat IgG from goat (GE Healthcare, NA935), EasyBlot anti-mouse IgG (HRP) (GeneTex; for reprobing IP membranes, also using GeneTex Easy Blocker solution); Alexa Fluor 488 chicken anti-mouse IgG (H+L) (Molecular Probes, A21200), Alexa Fluor 488 donkey anti-rat IgG (H+L) (Molecular Probes, A21208), Alexa Fluor 568 donkey anti-mouse IgG (H+L) (Molecular Probes, A10037), Alexa Fluor 647 chicken anti-rabbit IgG (H+L) (Molecular Probes, A21208) (1:500 IF), Alexa Fluor 488 and 568 phalloidin (Life Technologies, A12379, A12380); Hoechst 3342 (Life Technologies, H3570).

### Cell culture

All cells were cultured in a 37°C, 5% CO_2_ incubator. Parental MDCKII cells [from European Collection of Authenticated Cell Cultures (ECACC), operated by Public Health England] were maintained in Dulbecco's modified Eagle’s medium (DMEM, Invitrogen) in the presence of 10% foetal bovine serum (FBS, GIBCO). Cell lines were routinely tested for mycoplasma contamination by our in-house facility. MDCKII cells with dox-inducible expression of Myc-CASK, WT-Dlg1-HA, Δ66-Dlg1-HA and D66-HA were maintained in DMEM with 10% tetracycline-free FBS with G418 (1 mg/ml, Sigma-Aldrich) and puromycin (2 μg/ml, Sigma-Aldrich). Cells expressing constitutive knockdown pSuper plasmids were maintained in DMEM with puromycin (2 μg/ml, Sigma-Aldrich). Cells expressing inducible knockdown plasmids were maintained in DMEM with G418 (1 mg/ml). MDCKII cells expressing histone-2B-CFP and actin chromobody were grown in DMEM with 10% tetracycline-free FBS with G418 (1 mg/ml) and blasticidin (5 μg/ml, Sigma-Aldrich). MCF10A cells (a gift from Dr Gillian Farnie, University of Oxford, UK), were cultured in complete medium composed of whole minimal DMEM medium components supplemented with 100 ng/ml cholera toxin, 20 ng/ml epidermal growth factor, 10 μg/ml insulin and 0.5 μg/ml hydrocortisone.

### Cysts

Two methods for 3D culture were used in this paper. In the first 3D culture method, base layers (250 μl) of collagen I solution (2 mg/ml collagen I with added 1 M NaOH at 0.0023 times the volume of added collagen I) were set in 24-well plates (20 min, 37°C), before the addition of 300 μl collagen top layers containing 3.6×10^4^ cells in a single-cell suspension for lumen formation or spindle orientation assays, or 2×10^5^ cells for early spindle orientation assays. Gels were cultured in 700 μl medium for 1 day for early spindle orientation assays, 4–6 days for spindle orientation assays, and 8–10 days for lumen formation assays before immunostaining, with 50% of the media being replaced every 2–3 days.

Cyst-containing gels were washed twice in PBS, fixed in 3.7% formaldehyde for 15 min at room temperature, washed twice in PBS, carefully removed from the wells with tweezers and put into 1.5 ml Eppendorf tubes. Cysts were permeabilised in 0.5% Triton X-100/PBS for 30 min at room temperature, washed twice in PBS, incubated with blocking solution (10% FBS/PBS) for 1 h at room temperature, incubated with primary antibody in 2% FBS/PBS overnight at 4°C, washed in PBS for 6×30 min at 4°C, incubated with secondary antibody, phalloidin and Hoechst 33342 in 1% FBS/PBS overnight at 4°C, washed in PBS for 6×30 min at 4°C, and then mounted onto slides in mounting medium for imaging on the low-light, macro-confocal or SP8 confocal microscopes.

In the second 3D culture method, cells were trypsinised to a single-cell suspension at 1.5×10^4^ cells/ml in complete medium containing 2% Matrigel (BD Matrigel Matrix Phenol Red-Free, 10 ml, 356237). Suspensions (250 μl) were plated into 8-well chamber slides (Ibidi, 80826) and pre-coated with 10 μl of ice-cold 100% Matrigel. Cells were grown for 4 days before fixation in 3.7% formaldehyde for 15 mins at room temperature, permeabilised in 0.5% Triton X-100/PBS for 20 min, washed twice in PBS, incubated with blocking solution (10% FBS/PBS) for 1 h, and stained in wells with the process as above. A similar method was applied for the formation of MCF10A acini, using the same reagents and cell plating density. These acini were left to grow for 10 days in Matrigel culture before fixation in 3.7% formaldehyde and treatment as above. 50% of media was replaced every 2–3 days.

### Generation of cell lines

Plasmids were introduced into cells either by transfection using TransIT-LT1 (Mirus) according to the manufacturer's instructions or by retroviral transduction as previously described (Woodcock et al., 2010). For inducible overexpression, MDCKII were retrovirally transduced with pRetro-Tet-ON followed by selection with G418 (1 mg/ml, Sigma-Aldrich). pRetro-XT-based constructs were then retrovirally transduced and cells selected with puromycin (2 μg/ml, Sigma-Aldrich). For live imaging, cells were FACS-sorted to produce a population expressing both CFP and RFP markers. For inducible cell lines, clones of MDCKII cells expressing dox-inducible shRNAs to CASK, against two independent target sequences (CASK RNAi#1 and #2), were selected by single-cell sorting and screening for efficient knockdown of CASK upon addition of doxycycline.

### Plasmids and cloning

#### myc-CASK

The EcoRI myc-Cask fragment from myc-Cask-FL (a gift from Yi-Ping Hsueh, Institute of Molecular Biology, Academia Sinica, Taipei, Taiwan) was inserted into the pRetro-X-tight(puro) (Clontech), and mutated by Quikchange II mutatgenesis (Stratagene) to generate a sequence resistant to CASK shKD#1 shRNA and CASK siKD#1 siRNA sequences. The changes were as follows: wild-type sequence, 5′-TTAAGTACAGAAGATC-3′; resistant sequence, 5′-TTGAGCACCGGGGACC-3′. The following primers were used for the mutagenesis: forward, 5′-CATCAAGTCCAGGGTTGAGCACCGGGGACCTAAAGCGGGAAGC-3′; reverse, 5′-GCTTCCCGCTTTAGGTCCCCGGTGCTCAACCCTGGACTTGATG-3′.

#### D66HA

The N-terminal region of Dlg1 was amplified by PCR, and an HA tag added to the sequence of the reverse primer, along with restriction sites for insertion into the pRetro-X-Tight(puro) vector (Clontech). Correct insertion was verified by Sanger sequencing. Primers were: Dlg66 forward, 5′-TTTTGCGGCCGCATGCCGGTCCGGAAGCAA-3′; Dlg66 reverse, 5′-ACGTCTTAAGTCAAGCGTAATCTGGAACATCGTATGGGTATGGTTCACACTGCTTTGAATGA-3′.

#### WT-Dlg1 and Δ66-Dlg1

Full-length, HA-tagged WT-Dlg1 and the truncated Δ66-Dlg1 were amplified by PCR from a pCMV-SPORT6 vector (purchased from Open Biosystems) using the following primers: WT-Dlg1 forward, 5′-TTTTGCGGCCGCATGCCGGTCCGGAAGCAA-3′; Δ66-Dlg1 forward, 5′-TTTTGCGGCCGCATGTGTGTGGATCATTCAAAG-3′; Dlg1 reverse, 5′-AAAAAGAATTCTCAAGCGTAATCTGGAACATCGTATGGGTAAGCG-3′. Primers contained restriction sites for insertion into the pRetro-X-Tight(puro) vector (Clontech). Correct insertion was verified by Sanger sequencing.

#### Constitutive knockdown plasmids

shRNAs were screened for their CASK knockdown ability by transient transfection in HEK293T cells. Two sequences that successfully downregulated CASK (CASK iKD#1 and CASK iKD#2) were further sub-cloned into the pA′-TO dox-inducible RNAi vector (as previously described in Whalley et al., 2015). Additionally, the shRNA targeting sequence from CASK iKD#1 was cloned into the pRetro-Super (Clontech) vector, for constitutive knockdown of CASK. For Dlg1 knockdown, we used the sequence for siKD#3 against Dlg1 (from Awad et al., 2013) to produce primers to clone into pRetro-Super to generate Dlg1 shKD#1. Primers were as follows: CASK iKD#1 forward, 5′-GATCCCGTTAAGTACAGAAGATCTATTCAAGAGATAGATCTTCTGTACTTAACTTTTTGGAAA-3′; CASK iKD#1 reverse, 5′-AGCTTTTCCAAAAAGTTAAGTACAGAAGATCTATCTCTTGAATAGATCTTCTGTACTTAACGG-3′; CASK iKD#2 forward, 5′-GATCCCGCTCAGATGGAATGCTTTATTCAAGAGATAAAGCATTCCATCTGAGCTTTTTGGAAA-3′; CASK iKD#2 reverse, 5′-AGCTTTTCCAAAAAGCTCAGATGGAATGCTTTATCTCTTGAATAAAGCATTCCATCTGAGCGG-3′; CASK shKD#1 forward, 5′-GATCCGATAAGTACAGAACATCTATTTCAAGAGAATTCTTCT-3′; CASK shKD#1 reverse, 5′-AATTCAAAAAATTAAGTACAGAAGATCTATTCTCTTGAAATAGAT-3′; Dlg1 shKD#1 forward, 5′-GATCCCGATATCCTCCATGTTATTATTCAAGAGATAATAACATGG-3′; Dlg1 shKD#1 reverse, 5′-AGCTTTTCCAAAAAGATATCCTCCATGTAATAATCTCTTGAATAA-3′. The non-targeting (shRNA knockdown) plasmid used targets the sequence: 5′-ATGAAGTCGCATGGTGCAG-3′. The actin chromobody was purchased from ChromoTek. The histone-2B-CFP plasmid was the same as previously used in Whalley et al. (2015).

#### Transient transfection of siRNA

Transient silencing of CASK and Dlg1 was achieved by transfection of siRNA oligos from Eurofins MWG operon into MDCKII cells using Lipofectamine RNAiMax (Invitrogen, Life Technologies) according to the manufacturer's instructions. Cells were processed and analysed 48h post-transfection. siRNA sequences were as follows: CASK siKD#1, 5′-GGUUAAGUACAGAAGAUGUAATT-3′; CASK siKD#2, 5′-GGGAGUAUUACCUUCAAGAUUTT-3′; Dlg1 siKD#1, 5′-CAACTCTTCTTCTCAGCCTTT-3′; Dlg1 siKD#2, 5′-CAGAAGAACAAGCCAGAAATT-3′; sDlg1 siKD#3, 5′-GAUAUCCUCCAUGUUAUUATT-3′; siLuc (control) 5′-CGUACGCGGAAUACUUCGA-3′. Non-targeting siRNA sequence was Dharmacon siGENOME Non-Targeting siRNA #4.

### Protein analysis

Cells were lysed in immunoprecipitation (IP) lysis buffer [50 mM Tris-HCl, pH 7.5, 150 mM NaCl, 1% (v/v) Triton X-100, 10% (v/v) glycerol, 2 mM EDTA, 25 mM NaF and 2 mM NaH_2_PO_4_] containing a protease inhibitor cocktail (Sigma-Aldrich) and phosphatase inhibitor cocktails 1 and 2 (Sigma-Aldrich). For immunoprecipitation, lysates were incubated with the appropriate antibody pre-bound to 20 μl of GammaBind G Sepharose (Amersham) for 3 h at 4°C (for HA pull-down) or overnight (for endogenous immunoprecipitations), washed in IP lysis buffer, then eluted with 1×SDS-PAGE sample buffer (Nupage, Invitrogen).

### Immunofluorescence

For immunofluorescence in 2D, cells were grown on coverslips and fixed with either 100% ice-cold methanol for 5 min at −20°C, or 3.7% formaldehyde for 20 min at room temperature. Staining was performed by permeabilisation in 0.5% Triton X-100 in PBS (for formaldehyde-fixed samples) and blocking in 1% BSA/PBS for 1 h at room temperature before successive incubation with primary (overnight at 4°C) and then secondary antibodies. Coverslips were mounted to slides using Fluromount-G (Southern Biotech) along with Hoescht 33342 (1:5000) for nuclear staining.

For immunofluorescence in 3D of MDCKII cysts grown in collagen, the collagen plug was fixed in-well with formaldehyde for 20 min at room temperature, and washed three times with PBS. Cysts were permeabilised with 0.5% Triton X-100 in PBS for 20 min at room temperature with gentle rocking, before the whole collagen plug was gently placed in an Eppendorf tube, before being blocked with 2% BSA for one hour. Cysts were incubated with primary antibodies in 1% BSA/PBS overnight at 4°C with gentle rotation, before being washed six times for 30 min each time in PBS. Secondary antibodies were applied overnight in the same way, along with Hoescht 33342 (1:5000) for nuclear staining. The collagen plug was then mounted on a glass slide, covered with mounting media (Fluromount-G) and a coverslip gently placed on top.

For MDCKII cysts and MCF10A cysts grown in Matrigel, these were fixed in the wells of the chamber slide using formaldehyde (or ice-cold methanol for NuMA staining), as above, followed by blocking in 1% BSA for 1 h at room temperature, before staining overnight with primary antibodies in 1% BSA/PBS at 4°C. This was followed by three PBS washes for 30 min each at room temperature, before staining with secondary antibodies in 1% BSA/PBS overnight at 4°C. Cysts were washed three times in PBS before being left in 300 µl PBS for imaging.

### Microscopy

The spinning-disc confocal microscope was based around an Olympus IX81, using the Sedat filter set (Chroma, 89000), an array of imaging lasers (406, 488, 548, 645 nm) and an Apochromat ×100 1.45 NA oil objective. The low-light microscope was based around a Zeiss Axiovert 200 M, with an Andor iXon DU888+ camera, a 300 W xenon light source, using the Sedat filter set, and a Zeiss alpha Plan-Apochromat ×100 1.45 NA oil objective. The macro-confocal microscope was based around a Leica TCS LSI using a Leica Plan-Apochromat ×5 0.50 NA objective, an array of solid-state imaging lasers (488, 532 and 635 nm) and an ultrahigh dynamic photomultiplier. Images were captured using MetaMorph software (Molecular Devices) or Leica proprietary imaging software. The Deltavision Core (Applied Precision Instruments) system was based around an Olympus IX71 microscope with illumination achieved by white light LED and a 300 W xenon light source for fluorescence. The Sedat filter (Chroma, 89000) set was utilized for fluorescence imaging using an Olympus UPLSAPO 60XO 1.35 NA objective, and image capture was via a Roper Cascade II 512B EMCCD camera and SoftWorx software (Applied Precision Instruments). Images were also acquired on an inverted Leica TCS SP8 confocal microscope equipped with PMT and Hybrid (HyD) detectors, with the tunable white laser (WLL) for Alexa Fluor 488, Alexa Fluor 555 and Alexa Fluor 647, and 405 nm UV laser for Hoescht 33342. For 2D imaging, the ×63 1.45 NA oil immersion objective (Leica) was used, while for 3D imaging the ×25 0.95 NA water lens (Leica) was used. For 3D imaging we also used an upright Leica TCS SP8 confocal microscope equipped with PMT and Hybrid (HyD) detectors, and laser lines at 405, 488, 568 and 647. Images were captured in LAS AF (3.0.1) Leica software. Images were also acquired using a Zeiss Observer equipped with a Zeiss LSM 880 scan head with the AiryScan detector, with Argon laser 458, 488, 514 nm (Lasos, Jena, Germany), Diode 405-30 (Lasos), DPSS 561-10 (Lasos) and HeNe 633 nm (Lasos) were utilised for illumination and a Plan-Apochromat 40×/1.4 Oil (Zeiss) objective lens. All equipment control, acquisition and processing of AiryScan images was performed in Zen Black (Zeiss). All images were processed in ImageJ. Images in the same figure panel stained with the same antibody are all set to the same minimum and maximum brightness for comparison of localisation and intensity.

### Live imaging 3D culture

Live imaging was performed using cysts grown in optical bottom black 96-well plates (Thermo Scientific, 165305) on a layer of Matrigel, 5 µl per well (as for growth in 8-well chamber slides), with 2×10^4^ cells per well. Wells were filled to maximum volume with media supplemented with 2% Matrigel and imaged using an Opera Phenix (Perkin Elmer) with temperature and environmental controls (37°C and 5% CO_2_), using a ×20 NA 0.95 water immersion lens (Zeiss). Frames were captured at 7.5 min intervals for between 6 and 12 h. Movies were exported from the Columbus software and spindle orientation angles were analysed manually in ImageJ.

### Quantification of LGN and NuMA levels

From confocal stacks of mitotic cells, the fluorescence of LGN and NuMA at the membrane was calculated for each pole of the cell, in the stack where the fluorescence at the membrane was strongest, by manually drawing a region of interest in ImageJ along the membrane (line width: 5), and recording the mean intensity. A second line was drawn, adjacent to the first, and of similar length, in the cytoplasm immediately adjacent to the membrane, and the intensity recorded. The ratio of membrane:cytoplasm intensity was then calculated as (membrane−cytoplasm)/(cytoplasm) (which normalises for local variations in intensity between cells, and gives a negative value where the cytoplasmic reading is higher than the membrane reading) in Microsoft Excel and imported into Prism for analysis.

### Quantification of astral microtubule intensity

Maximal projections of spinning disc confocal stacks were generated using ImageJ. Two regions of interest were drawn, encompassing the mitotic spindle (*a*) and the cell area containing astral microtubules (*b*). The formula (*b*−*a*)/*a* was used to calculate the relative astral microtubule intensity, normalised to the intensity of the mitotic spindle.

### Quantification of spindle orientation

For spindle orientation in 3D culture, confocal or widefield images of the dividing cell were manually analysed using the Measure Angle tool in ImageJ, with a line drawn through the axis of the dividing cell, and the angle measured to the nearest apical surface. For 2D spindle orientation, the *xyz* coordinates of the centre of each centrosome (marked by pericentrin staining) were determined by marking the appropriate stacks when viewed in ImageJ. Standard trigonometric functions were used to calculate the angle relative to the surface of the microscope slide.

### Statistical analysis

Statistical differences between data were analysed in Prism (GraphPad) software using appropriate statistical tests (with adjustments for multiple comparisons where appropriate); absolute *P*-values and the individual tests used are specified in figure legends.

## Supplementary Material

Supplementary information
