# Supplementary material for: The interaction between CASK and the tumour suppressor Dlg1 regulates mitotic spindle orientation in mammalian epithelia
Source: J Cell Sci. 2019 Jul 15;132(14):jcs230086. doi: 10.1242/jcs.230086 (PMC6679578; doi:10.1242/jcs.230086)
Supplement: Supplementary information [file joces-132-230086-s1.pdf]

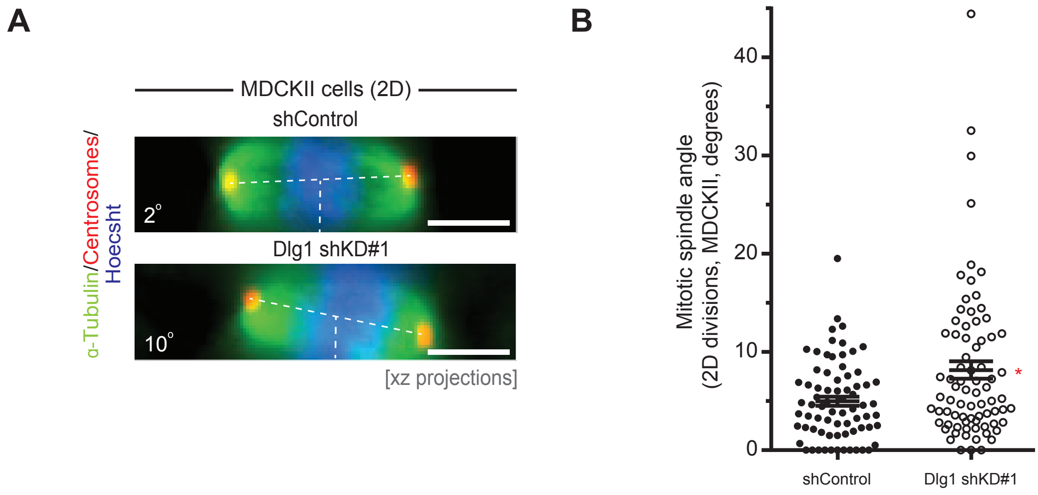

Figure S1

A) Representative images of XZ projections from MDCKII cells grown in 2D, showing tilted mitotic spindles at metaphase after constitutive Dlg1 knockdown (Dlg1 shKD#1), and annotated to show guide lines and spindle angles. B) Quantification of spindle angles from MDCKII Control (Non-Targeting) and Dlg1 knockdown cells grown in 2D, n=73/77 from three independent experiments; * p= 0.033 (Kruskal-Wallis test). Error bars show mean +/- SEM.

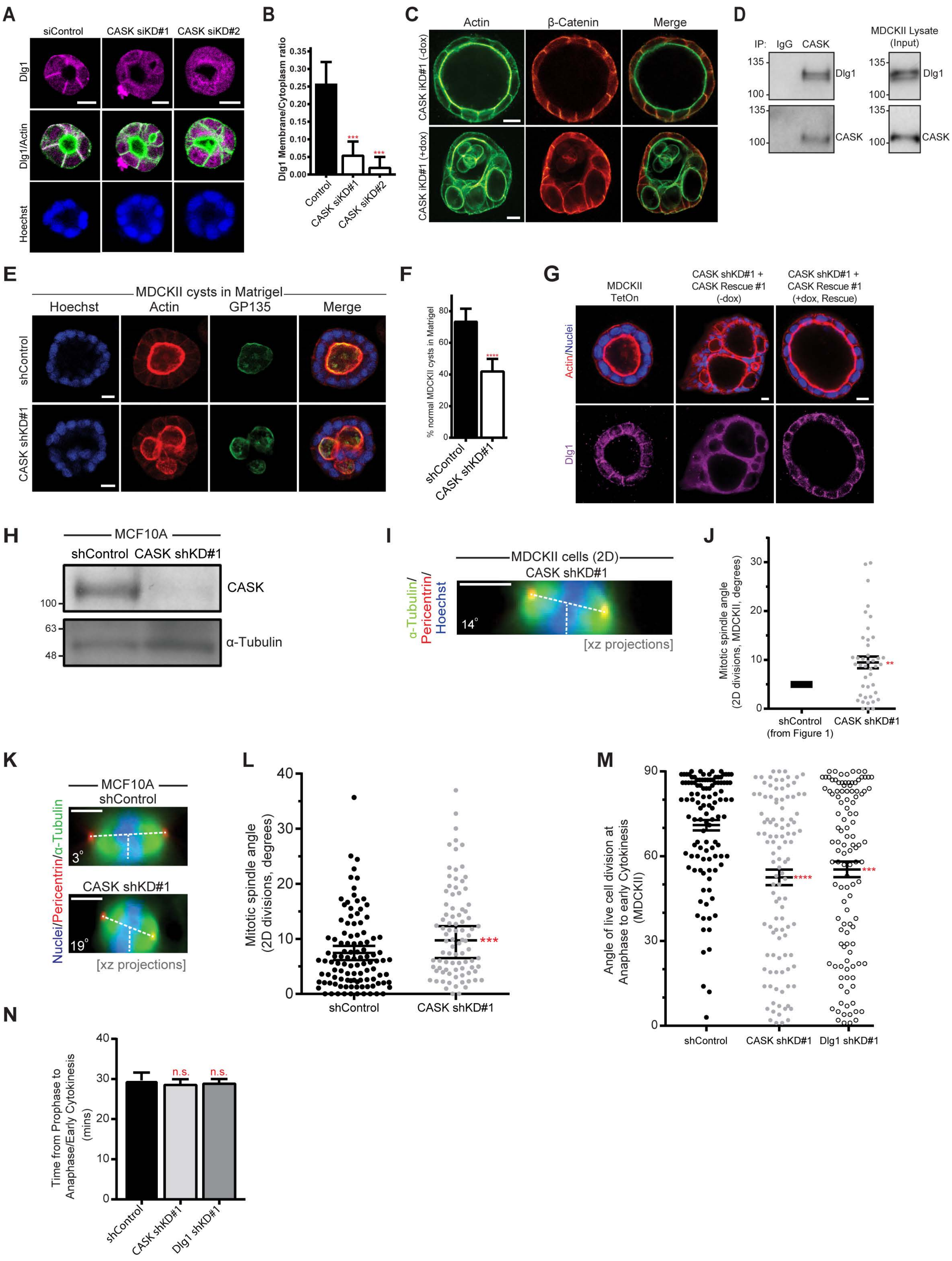

Journal of Cell Science • Supplementary information

Figure S2

A) Representative confocal images of MDCKII cysts in Matrigel showing membrane localised Dlg1 (magenta) which is lost following CASK knockdown. B) Quanti�ication of Dlg1 membrane intensity from (A), n=131/99/59 cell junctions measured across 4 independent experiments. *** p= 0.0005 and 0.0003 for CASK siKD#1 and CASK siKD#2 respectively, One-way Anova. C) Confocal images of MDCKII cysts grown in Collagen I showing strong basolateral staining of β-Catenin in both Control (-dox) and CASK iKD#1 (+dox). D) Western blot showing the endogenous CASK and Dlg1 interaction in MDCKII cells, following CASK immunoprecipitation. E) Confocal images of cysts from MDCKII cells transfected with either Control (Non-Targeting) or CASK shKD#1, grown in 2% Matrigel and displaying either a normal (top panels) or a multilumen phenotype (bottom panels). F) Quantification of cysts with normal lumens as depicted in (E), N=3 independent experiments, more than 100 cysts examined per experiment; **** p=2.6x10-5 (unpaired Student's T-test, 2 tailed). G) Confocal images of MDCKII cysts showing basolateral localisation of Dlg1 in Control cysts (MDCKII Tet-On, bottom panel), loss of Dlg1 lateral staining following CASK knockdown (-dox, middle panel) and restoration of basolateral Dlg1 staining upon CASK re-expression (+dox, bottom panel). H) Western blot showing depletion of CASK in MCF10A cells expressing CASK shKD#1. I) Representative image of XZ projections from MDCKII cells grown in 2D, showing tilted mitotic spindles at metaphase after CASK knockdown, and annotated to show guide lines and spindle angles. J) Quantification of spindle angles from MDCKII Control (Non-Targeting) and CASK knockdown cells grown in 2D as in (I), n=42 from three independent experiments; ** p= 0.0046 (Mann-Whitney test), compared with control (control mean and SEM are shown as in Supplementary Figure 1B). Error bars show mean +/- SEM. Data analysed using Kruskal-Wallis test. K) XZ projection of metaphase MCF10A cells showing tilting of the metaphase spindle after knockdown of CASK (bottom panel). Scale bar is 5 μm. L) Quantification of 2D spindle angle in MCF10A cells from experiments as in (K). n=107/88 cells pooled from three independent experiments; *** p=0.00052 (Mann-Whitney test). Error bars show mean +/- SEM. M) Quantification of cell division angles from live imaging experiments, only from cells in anaphase, telophase or early cytokinesis (n=112/110/116 cells from three independent replicates); **** p=2.9x10-6, *** p=0.00031. Values are mean +/- SEM (Kruskal-Wallis test.) N) In cases where individual cell divisions could be followed from prophase through to anaphase/telophase, the timing of progression was recorded (interval between images was 7.5 minutes). Graph shows mean, error bars show 1SD (n=51/30/24 from three biological replicates). n.s. = not significant (p= 0.9134 and p= 0.9653 for CASK and Dlg1 shRNA respectively). (One-way Anova.)

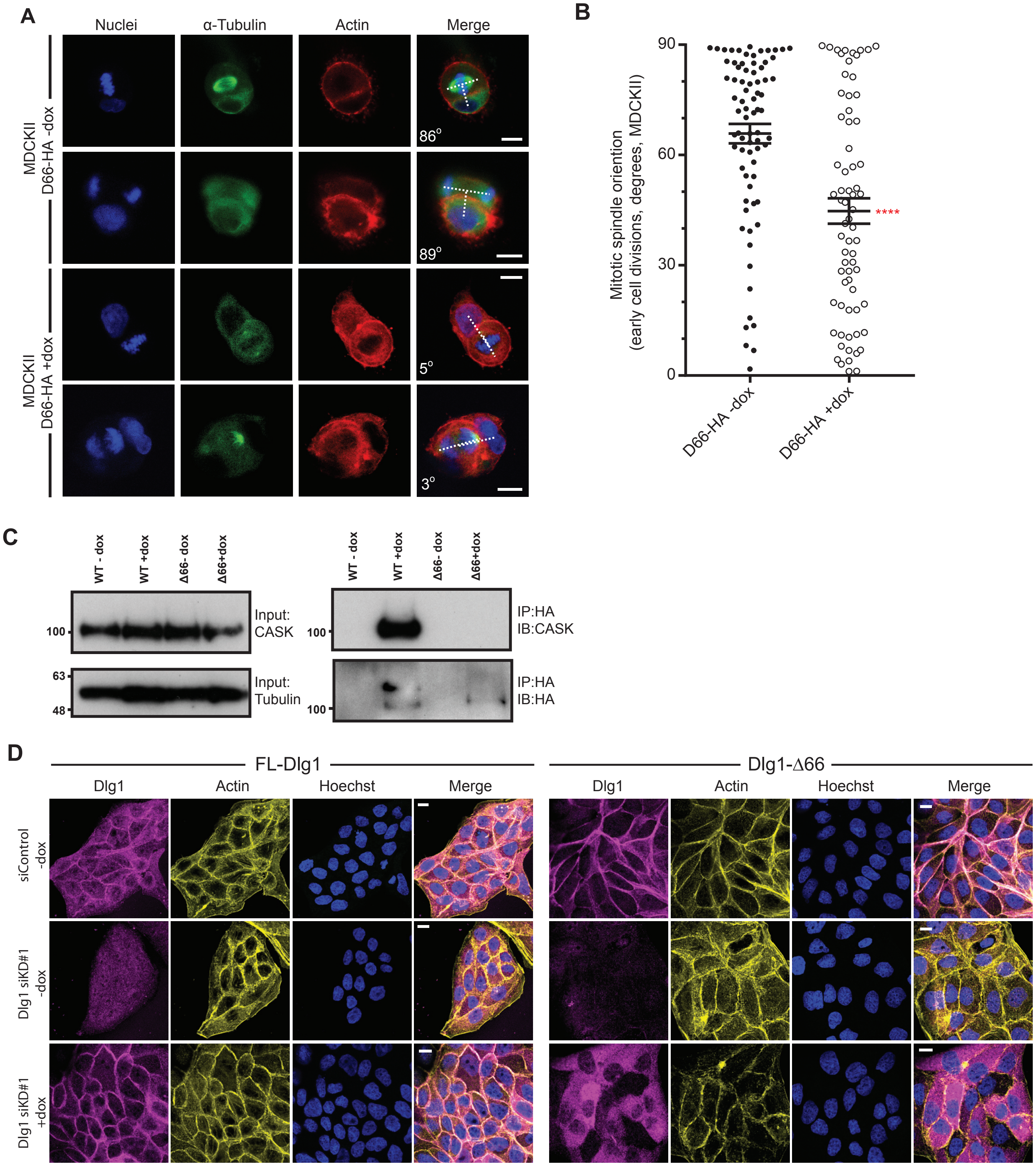

Figure S3

A) Example images of early cell divisions in MDCKII cysts showing misoriented divisions following expression of D66-HA. B) Quantification of spindle orientation angles in early MDCKII cysts with and without D66-HA expression as in (A), n=76/69 from three independent experiments; **** p=2.3x10-5 (Mann Whitney Test). Error bars show mean +/- SEM. C) Western blot showing detection of an interaction between exogenous WT-Dlg1 and endogenous CASK, but not between Δ66-Dlg1 and endogenous CASK, in MDCKII cell lysates. D) Representative confocal images showing Dlg1 intensity and localisation in control (siLuc) and Dlg1-depleted cells (Dlg1 siKD#1), expressing either WT-Dlg1 or Δ66-Dlg1 (+dox). WT-Dlg1 localises to the cell cortex, whereas Δ66-Dlg1 is primarily cytoplasmic.

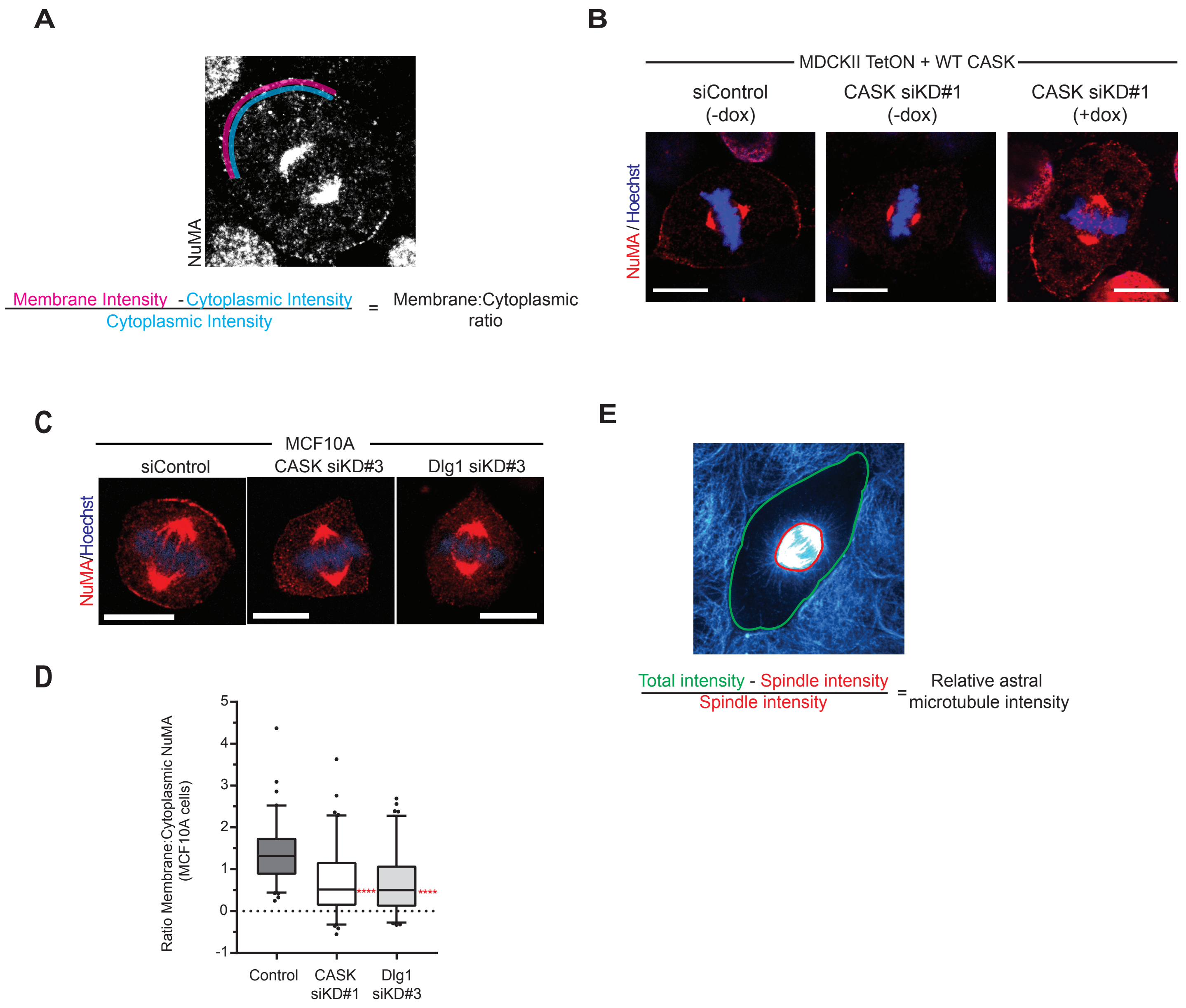

Figure S4

A) Schematic of Membrane:Cytoplasmic ratio quantification used for LGN and NuMA imaging; purple line represents membrane intensity quantification, blue line represents cytoplasmic intensity quantification. B) Representative confocal images of NuMA staining in MDCKII cells inducibly expressing siRNA-resistant CASK upon addition of dox (+dox) treated with either siControl (Non-Targeting siRNA) or CASK siKD#1. C) Representative confocal images of NuMA staining in siControl MCF10A cells (Non-Targeting siRNA), with reduced membrane-associated NuMA in CASK and Dlg1 knockdown MCF10A cells (CASK siKD#1 and Dlg1 siKD#3). D) Quantification of Membrane:Cytoplasmic NuMA ratio. n=84/81/88 membrane measurements from three independent experiments; **** p=3.7x10-7 and 9.0x10-9 (one-way Anova) for CASK siKD#1 and Dlg1 siKD#3 respectively. Box shows 25-75 percentile marked with the median, whiskers show 5-95 percentile, dots represent measurements outside this range. E) Schematic of method for calculating astral microtubule intensity from spinning disc confocal images of metaphase cells.

Journal of Cell Science • Supplementary information

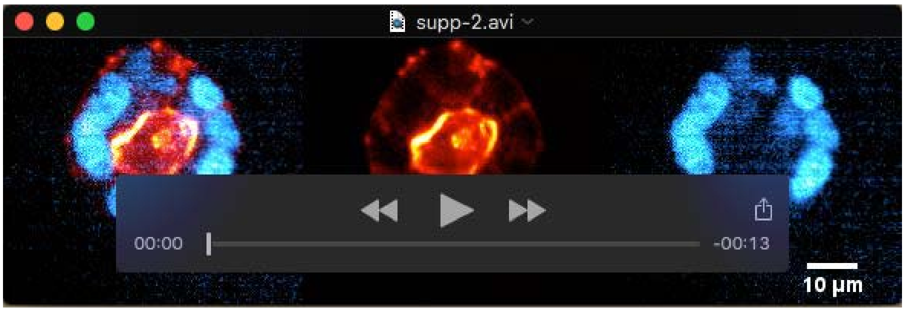

Movie 1

Non-Targeting shRNA-expressing control cyst, grown on Matrigel, showing normally oriented cell divisions; cell division starting at 0 minutes corresponds to division in Figure 2M. Other divisions in the movie are also oriented in the plane of the epithelium. Left panel – merged image. Middle panel – Actin Chromobody, tagRFP. Right panel – Histone-2B-CFP. Right panel – Histone-2B-CFP. 4 frames/second.

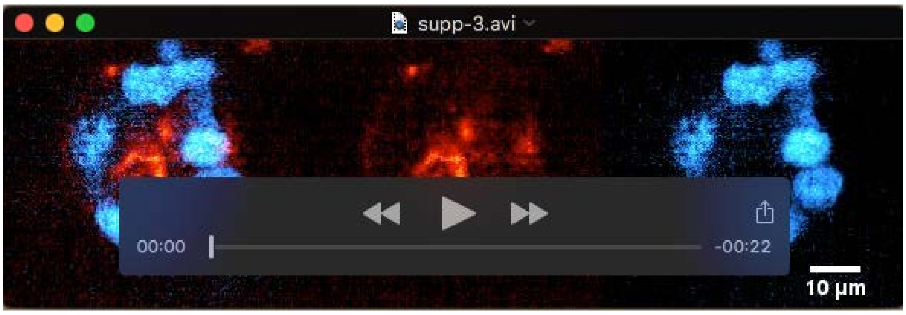

Movie 2

CASK shKD#1 cyst, grown on Matrigel, showing a number of abnormally oriented cell divisions; cell division starting at 0 minutes, corresponds to division in Figure 2M. Other divisions in the movie are also abnormally oriented, and what appears to be a new lumen forms towards the end of the recording. Left panel – merged image. Middle panel – Actin Chromobody, tagRFP. Right panel – Histone-2B-CFP. 4 frames/second.

Journal of Cell Science • Supplementary information

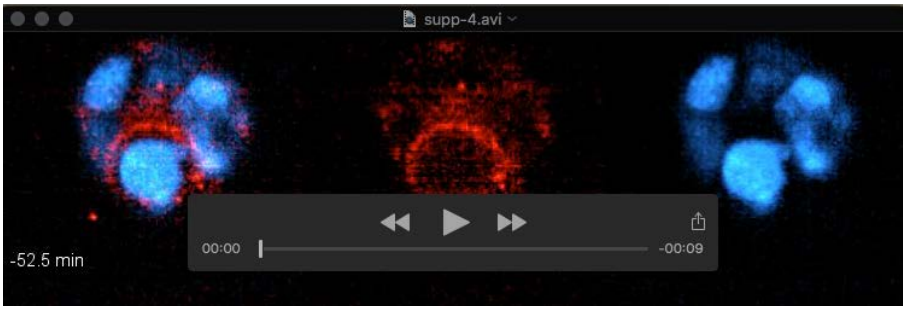

Movie 3

Dlg1 shKD#1 cyst, grown on Matrigel, showing an abnormally oriented cell division starting at 0 minutes, corresponds to division in Figure 2M. Left panel – merged image. Middle panel – Actin Chromobody, tagRFP. Right panel – Histone-2B-CFP. 4 frames/second.
